# Enhanced Photoluminescence in Acetylene-Treated ZnO Nanorods

**DOI:** 10.1186/s11671-016-1627-y

**Published:** 2016-09-20

**Authors:** Luke Jäppinen, Tero Jalkanen, Brigitte Sieber, Ahmed Addad, Markku Heinonen, Edwin Kukk, Ivan Radevici, Petriina Paturi, Markus Peurla, Mohammad-Ali Shahbazi, Hélder A. Santos, Rabah Boukherroub, Hellen Santos, Mika Lastusaari, Jarno Salonen

**Affiliations:** 1 Department of Physics and Astronomy, University of Turku, FI-20014 Turku, Finland; 2 Unité Matériaux et Transformations, Université Lille 1, 59655 Villeneuve d’Ascq, France; 3 Institute of Biomedicine, University of Turku, FI-20014 Turku, Finland; 4 Division of Pharmaceutical Chemistry and Technology, Faculty of Pharmacy, University of Helsinki, FI-00014 Helsinki, Finland; 5 Institut d’Electronique, de Microélectronique et de Nanotechnologie (IEMN), Université Lille 1, 59652 Villeneuve d’Ascq, France; 6 Department of Chemistry, University of Turku, FI-20014 Turku, Finland

**Keywords:** ZnO, Photoluminescence, C_2_H_2_, Nanorods, Thermal annealing

## Abstract

Zinc oxide (ZnO) nanorods were manufactured using the aqueous chemical growth (ACG) method, and the effect of thermal acetylene treatment on their morphology, chemical composition, and optical properties was investigated. Changes in the elemental content of the treated rods were found to be different than in previous reports, possibly due to the different defect concentrations in the samples, highlighting the importance of synthesis method selection for the process. Acetylene treatment resulted in a significant improvement of the ultraviolet photoluminescence of the rods. The greatest increase in emission intensity was recorded on ZnO rods treated at the temperature of 825 °C. The findings imply that the changes brought on by the treatment are limited to the surface of the ZnO rods.

**Electronic supplementary material:**

The online version of this article (doi:10.1186/s11671-016-1627-y) contains supplementary material, which is available to authorized users.

## Background

Zinc oxide (ZnO) is a versatile, multipurpose oxide semiconductor with applications ranging from paints and sunscreens to thin-film transistors and gas sensors [1–3]. Small-scale ZnO structures, such as nanorods, nanorings, and nanowires, are of special interest, since ZnO is known to exhibit a particularly rich spectrum of such morphologies [4, 5]. This abundance of nanostructures creates a wide range of possibilities for ZnO-based devices. ZnO is biocompatible, which extends its possibilities into biological applications [6]. As an example, ZnO nanorods have been utilized to manufacture biosensors for glucose detection [7]. Considerable interest has also arisen as a result of reports on room-temperature ferromagnetism in doped ZnO, as predicted by Dietl et al. [8] for Mn-doped ZnO and observed by several research groups, first using metallic dopants and later even with nonmetallic ones, especially carbon [9–11].

As a direct bandgap compound semiconductor, ZnO is of considerable interest in the field of optoelectronics [4]. Like any semiconductor material, the photoluminescence properties of ZnO are directly linked to its energy level structure. Thus, changes in the photoluminescence emissions resulting from treatments provide a way of evaluating how these treatments could be used to modify and improve the photoelectric performance of ZnO-based devices. They may also be of importance when considering light-based sensor applications for the material. One way to modify the photoelectric properties of the material is doping using different materials. As mentioned, carbon doping has previously been pursued as a method to promote ferromagnetism in ZnO. Understanding the effects of carbon doping is important not only in order to understand the magnetic phenomena but also because of the popularity of organic chemical methods, which allow the synthesis of nanostructures in low temperatures [12–14]. Such methods naturally involve the possibility of residual organic impurities in the resulting materials.

Many carbon doping schemes involve a solid-state step resulting in carbon-doped bulk ZnO (usually powder) [15–17]. However, this approach can be problematic when creating ZnO nanostructures, because it involves applying an additional reactant in the process which may interfere with the formation of nanostructures. In an earlier paper, we reported room-temperature ferromagnetism in ZnO nanorods manufactured using a chemical bath deposition method and subsequently thermally treated using acetylene gas [18]. Using this method, the fabrication of nanorods and carbon doping are separated into two different processes, increasing the versatility of the ZnO nanostructure synthesis process.

In the current study, ZnO nanorods were grown using the aqueous chemical growth (ACG) method introduced by Vayssieres in order to elucidate the effect of the growth method on the properties of acetylene-treated ZnO [19]. Low-temperature chemical methods are widely used to synthesize ZnO nanostructures and thin films thanks to their ease of use and generally benign chemicals involved [20–24]. The ACG process applied here is especially attractive thanks to it being both inexpensive and easily scaled to industrial levels [25]. The treated nanorods have been investigated using various methods to gain insight on the optical properties of the material and the changes induced by the treatment.

## Methods

A chemical method to manufacture ZnO nanorods, based on Vayssieres’ ACG method [26], was used. To synthesize ZnO nanorods, an equimolar aqueous solution of zinc nitrate (Zn(NO_3_)_2_⋅6H_2_O, Sigma-Aldrich) and hexamethylenetetramine (HMT, (CH_2_)_6_N_4_, Merck) was prepared. Deionized (DI) water (MilliQ, 18.2 MΩ cm) was utilized as a solvent. All chemicals were analytical grade and used without further purification. After testing molar concentrations between 0.001–0.5 M, most samples were made using 0.005 M and 0.01 M solutions, as this molar range was found to provide the most uniform and reproducible layers of rods. Substrates of *p*-type <100 > silicon (Si) wafers (Siegert Wafer, resistivity 0.01–0.02 Ω cm) were cleaned and placed in the solution. The cleaning process consisted of sonication of the Si substrates first in DI water and then in acetone for 280 s. The substrates were placed polished side facing upwards in 20 ml glass ampoules filled with the solution and put in an oven at a temperature of 95 °C overnight (20 h). This time was found to be optimal for producing high-quality layers of rods. After treatment, the substrates were cleaned with copious amounts of DI water to remove the excess solution and trace substances. Cleaned samples were allowed to dry in air.

The treatment of samples was done using acetylene gas, and the method is also described in detail in [18]. In the current work, the samples were placed in a quartz tube and flushed with 1 l/min nitrogen gas in room temperature. Next, a 14 min 45 s acetylene/nitrogen flush (2 l/min, 1:1 by volume) at room temperature was used. Acetylene flow was cut, and after allowing for excess acetylene to clear for 15 s, a 10-min thermal treatment in a tube furnace under 1 l/min N_2_ flow was performed. For reference, some samples were thermally annealed, using the same process flow but without adding acetylene.

The samples were investigated with scanning (SEM, FEI Quanta 250 operated at 10 kV) and transmission electron microscopes (TEM, JEM-1400 Plus). For TEM and electron diffraction studies, the ZnO rods were dispersed on holey carbon support films and imaged with a TEM operated at 120 kV. Diffraction patterns were indexed using simulations from WebEMAPS software [27]. Additional TEM and high-resolution TEM (HRTEM) images were recorded on a CM30 Philips system operating at 300 kV.

X-ray diffraction (XRD) measurements were done using a Philips (currently Panalytical B.V., Almelo, Netherlands) X’Pert Pro X goniometer equipped with a *θ*/2*θ* diffractometer and a proportional counter detector. Cu Kα radiation was used, with the X-ray tube run at 40 kV/50 mA. The incident beam optics comprised of a 0.04-rad Soller slit, a 15-mm mask and a (1/4)° divergence slit. The diffracted beam optics consisted of another 0.04-rad Soller slit and a Panalytical Pixcel 1D multichannel detector with a nickel filter. Diffraction results were fitted using the MAUD software version 2.43 [28].

X-ray spectroscopy (XPS) measurements were performed using a PHI 5400 ESCA spectrometer (Perkin Elmer, USA) with a monochromatic Al K_α_ X-ray source (1486.7 eV). Survey spectra were collected using the pass energy of 89.45 eV and high-energy multiplex spectra of the main elements (C 1 s, O 1 s, Si 2p, and Zn 2p3) with the pass energy of 71.55 eV. Fitting was made using the XPSPEAK 4.1 software using 100 % Gaussian peak shape and increasing the number of peaks until *χ*^2^ values were less than 2⋅10^-3^.

Absorption spectra were recorded in the wavelength range 400–800 nm using a Perkin Elmer Lambda UV/Vis 950 spectrophotometer in polystyrene cuvettes with an optical path of 10 mm. The reflectance spectra were recorded using the Angle Absolute Universal Reflectance Accessory (URA) purchased from Perkin Elmer.

Photoluminescence spectra of the ZnO rods were studied in the wavelength range from 350 to 800 nm at room temperature using laser excitation and simultaneous detection of emitted light. An Oriel MS257 monochromator (with spectral resolution better than 1.3 nm in this spectral range), a NL100 nitrogen pulse laser with a wavelength of 337.1 nm (3.68 eV, 3.4 mW average power) and Hamamatsu R943-02 photomultiplier were used for measurements. For the steady-state measurements, an SRS 250 Boxcar Averager was used to integrate the response of the photomultiplier tube in a 9-μs range with a 1-μs delay after each laser pulse.

Excitation spectra were recorded using a Varian Cary Eclipse spectrophotometer. A xenon flash lamp was used for excitation, and the device was operated in simultaneous emission/measurement mode. A 5-nm excitation and a 10-nm emission slit were used.

Micro Raman spectroscopy measurements were conducted using a visible Labram HR spectrometer and a UV Labram HR (Horiba). In the visible range, the Raman backscattering was excited with an excitation wavelength of 532 nm. The beam was focused on the sample surface through an optical objective (6100, 0.9 NA) with a lateral resolution (XY) of less than 1 mm. For UV Raman analysis, the Raman backscattering was exited with a 266-nm laser line. A 680 (0.55 NA) objective was used that allowed to reach a measured lateral resolution (XY) of 0.5 mm. The spectral resolution was better than 2 cm^−1^ in both cases.

## Results and Discussion

### Electron Microscopy and Selected Area Diffraction

**Fig. 1:**
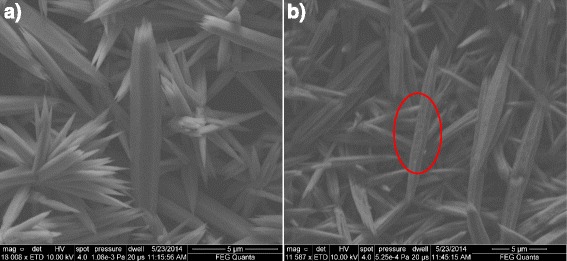
SEM micrographs of ZnO nanorods annealed in nitrogen (**a**) and treated with C_2_H_2_ (**b**)

SEM images of the investigated ZnO rods are shown in Fig. 1. It can be seen that the rods are arranged into clusters, with individual rods having a hexagonal shape, indicative of wurtzite ZnO [29]. In the absence of a seed layer on the substrate, the rods align randomly, and the smaller rods tend to form star-like clusters. Judging from the SEM images, the rods are between 500 nm and 1 μm in thickness and, on average, around 10 μm in length, although some longer rods are as long as 20 μm. The N_2_-annealed (annealing temperature 925 °C) rods, as shown in Fig. 1a, can be seen to have a smooth surface, which is the case in untreated rods as well. In contrast, the acetylene-treated rods (Fig. 1b) have a rougher surface, and the treatment creates some holes and grooves in them. However, unlike in the previous work, the rods are not completely eroded [18]. The grooves seem to preferentially run along the rods.

The length and diameter of the obtained rods agree with those reported by Vayssieres [26], though ours are not preferentially aligned perpendicular to the substrate. This may be due to different orientation or pre-processing of the Si surface (not divulged in [26]). Better alignment could be obtained by utilizing a ZnO seed layer [14, 30, 31].

**Fig. 2:**
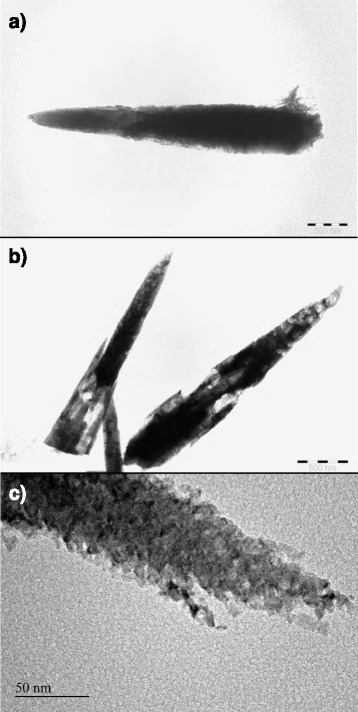
TEM micrographs of untreated (**a**) and acetylene-treated (**b**, **c**) ZnO nanorods. Scale bar 500 nm (**a**, **b**)/50 nm (**c**)

TEM micrographs of the rods are shown in Fig. 2. A clear difference between untreated (Fig. 2a) and acetylene-treated (Fig. 2b) rods can be observed. Large zones of low contrast, as seen in the latter picture, could be seen in acetylene-treated rods. We could not find such zones among the untreated rods. Together with SEM observations, we assume these zones to represent eroded areas of the ZnO nanorods. Some N_2_-annealed rods had similar zones but in very rare cases. Even when such zones were observed, the zones were distinctively smaller than in any of the acetylene-treated rods. A close-up look in the TEM image (Fig. 2c, taken with CM30 Philips) shows that the surfaces of the rods are made up of small nanoparticles, around 6–8 nm in diameter. Judging from the overall shape of the rods and the sharpness and intensity ratios of the observed XRD peaks (XRD results discussed later), it seems likely that the rods still contain a single-crystal core, as a rod made up entirely of smaller particles would be unlikely to retain the characteristic hexagonal shape.

**Fig. 3:**
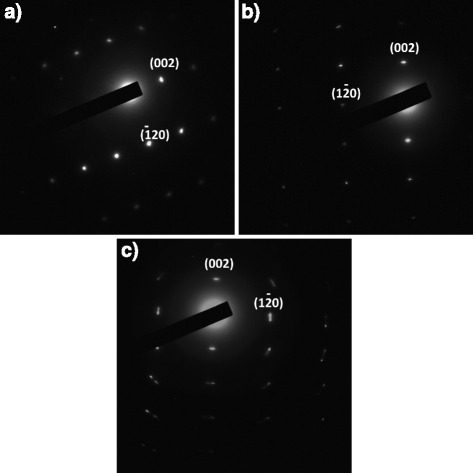
Selected area diffraction of untreated (**a**), N_2_-annealed (**b**), and acetylene-treated ZnO nanorods (**c**)

In Fig. 3, we show the selected area electron diffraction (SAED) patterns for untreated (a), N_2_-annealed (b), and acetylene-treated (c) ZnO nanorods. As-grown and nitrogen-annealed samples can be seen to display well resolved spots further confirming the existence of a crystalline wurtzite phase. The acetylene-treated samples display somewhat broadened spots, which may be the result of texturing and/or the increased roughness of the surface as a result of the erosion seen in the micrographs.

### X-ray Diffraction

**Fig. 4:**
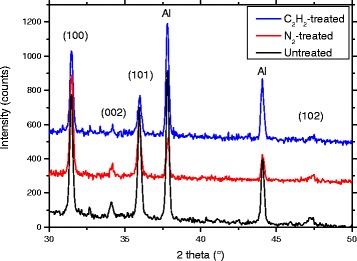
XRD diffractograms of acetylene-treated, N_2_-annealed, and untreated ZnO samples

XRD diffractograms of samples grown in a 20-h growth in 0.005 M solution are shown in Fig. 4. The treatment temperature was 925 °C. The XRD patterns show only the hexagonal wurtzite structure of ZnO identified through the characteristics peaks at 2*θ* angles of 31.4°, 34.1°, 35.9°, and 47.4° [32]. No other phases apart from the Al peaks, originating from the sample holder, are detected. In contrast to the ZnO rods investigated previously [18], neither annealing nor treating with acetylene has a large effect on the XRD patterns of the rods. No clear correlation between the treatment and the full width at half maximum (FWHM) of ZnO-related peaks is observed.

In the aforementioned study, acetylene treatment at 925 °C resulted in a total loss of XRD peaks due to erosion of the rods, which differs considerably from the behavior of the rods studied here. Possible reasons for this may lie either in differences in acetylene adsorption dynamics or, alternatively, different defect densities in the as-grown rods. The photoluminescence results (discussed later) show that the ACG rods contain significantly larger amounts of defects, yet erode significantly less than the earlier rods. While one might expect that more numerous defects should lead to increased susceptibility to acetylene-induced erosion, the results show that the relation between defect density and reactivity with acetylene is not straightforward. The results show that the response of seemingly identical ZnO nanostructures to acetylene treatment is heavily dependent on the synthesis method, which must be borne in mind when considering possible applications.

### X-ray Photoelectron Spectroscopy

**Fig. 5:**
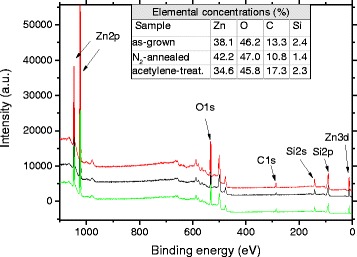
XPS spectra from as-grown (*red*), N_2_-annealed (*black*), and acetylene-treated (*green*) ZnO. The *inset* shows the elemental concentrations

The X-ray photoelectron spectroscopy (XPS) results from three ZnO nanorod samples are displayed in Fig. 5, and the concentrations of elements calculated from the spectra are shown in the inset of Fig. 5. The intensities have been shifted for clarity. There was no evidence of new peaks in any of the samples as a result of the treatment. An increase of 4.0 at.% in carbon concentration in the acetylene-treated sample was detected, while a reduction of 2.5 at.% in the N_2_-annealed sample was observed. One must keep in mind that adventitious carbon makes direct comparison of carbon concentrations measured by XPS somewhat unreliable. The amount of carbon detected in these measurements is roughly equivalent to that observed in the as-grown samples in the previous study [18, 33]. Part of the excess oxygen (in relation to zinc) is likely to originate from adventitious carbon species, but the contribution to the total amount of detected oxygen should be much less than the fractional amount of carbon detected, as adventitious carbon is generally comprised of hydrocarbons with only some oxidization, nor can the presence of silicon dioxide account for all the excess oxygen detected [34]. We may thus conclude that the surfaces of the rods contain at least some extra oxygen compared to stoichiometric ZnO.

The XPS results support the view that the nanorods investigated here behave very differently from the ones grown by chemical bath deposition in our previous study, where the C content in samples treated with acetylene in 925 °C increased from 12.3 to 78.1 at.% and the detected Zn content dropped from 43.3 at.% to zero [18]. However, in the ACG samples studied here, the changes are much more subtle. The ratio of zinc to oxygen is less than 1:1 in all samples but clearly lowest in the acetylene-treated sample. It is thus unlikely that zinc interstitials or oxygen vacancies be present in the samples in great numbers, especially after annealing. We also see no shifting in the Zn Auger peaks, ruling out the formation of clusters of metallic zinc, which could lead to ferromagnetism [35].

**Fig. 6:**
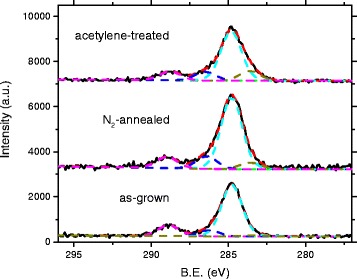
Deconvoluted C1s peaks from the XPS spectra

Curves fitted to the C1s peak can be seen in Fig. 6. Voigt profiles with 10 % Gaussian and 90 % Lorentzian weights and a Tougaard background have been used. The spectra have been calibrated by setting the prominent adventitious carbon C-C peak to 284.8 eV. Four peaks were fitted to the C1s feature: in addition to the adventitious carbon peak, one around 283.6 eV that is assigned to carbon bound to Zn, second near 286.5 eV which is assigned to a zinc oxycarbide complex and a fourth, clearly separate one around 288.8 eV that is associated with C-O bonds [15]. The positions and relative areas (in relation to all peaks of the spectrum) are presented in Table 1. The relative area of the C-C peak varies between 7.6 % (N_2_-annealed sample) to 11.1 % (acetylene-treated), giving an estimate of the effect of adventitious carbon contamination. The Zn-C peak relative area increases from 0.2 % in the as-grown sample to 0.7 % in the nitrogen-annealed one and 2.2 % in the acetylene-treated sample. The small increase with annealing is likely due to organic impurities bonding with zinc as a result of annealing, combined with a reduction of 2.1 to 1.2 % in the C-O peak.

**Table 1:** XPS C1s fitted peak positions and relative areas

Peak	1 (C-O)		2 (oxycarb.)		3 (C-C)		4 (Zn-C)	
Sample	Position (eV)	Relative area (%)	Position (eV)	Relative area (%)	Position (eV)	Relative area (%)	Position (eV)	Relative area (%)
As-grown	288.9	2.1	286.3	1.2	284.8	9.8	283.4	0.2
N_2_-annealed	289.0	1.2	286.4	1.3	284.8	7.6	283.6	0.7
Acet.-treat.	288.8	2.0	286.5	2.0	284.8	11.1	283.6	2.1

The O1s (see Additional file 1: Figures S1–S3) peak could be fitted with three peaks, in a manner described by Hsieh et al., with the highest energy (O_c_) peak corresponding to OH species on the surface [36]. While the lowest energy peak, related to O^2−^ ions in the wurtzite structure, is most pronounced in the N_2_-annealed sample, all samples have an O_c_ peak with comparable size, which makes it unlikely that the disappearance of the visible luminescence could be mainly attributed to the decomposition of Zn(OH)_2_ on the surface.

### Optical Properties

**Fig. 7:**
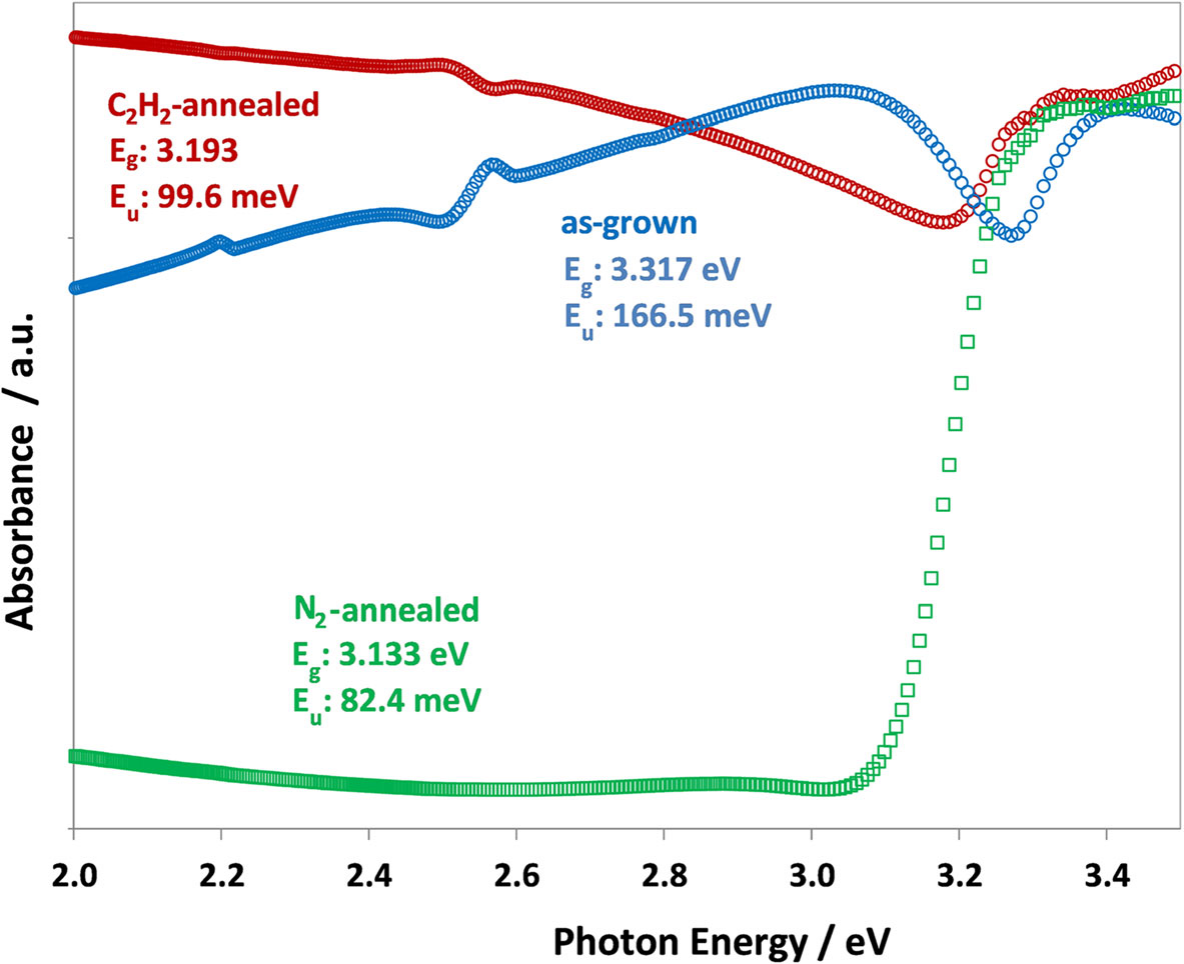
Absorbance (in reflectance mode) of ZnO rod samples. The gap and Urbach energies are shown

Figure 7 shows the absorbance spectra measured from ZnO samples along with their gap and Urbach energies, calculated from Urbach fits to the absorption edge. It can be seen that the as-grown sample displays an energy gap over 0.1 eV larger than the thermally treated samples. The Urbach energy, 166.5 meV, is also much larger than in the treated samples, which show Urbach energies of 82.4 and 99.6 meV for the N_2_-annealed and acetylene-treated samples, respectively. A larger Urbach energy is associated with greater lattice disorder in the ZnO lattice, which is likely to be greater in unannealed samples, thus leading to an increase in the Urbach energy [37]. The as-grown sample shows strong absorbance below the actual bandgap at 3.0 eV, which we attribute to a large concentration of defects present in the sample, evident from the luminescence properties (discussed below), and possible trace amounts of unreacted raw reagents on the surface of the rods.

**Fig. 8:**
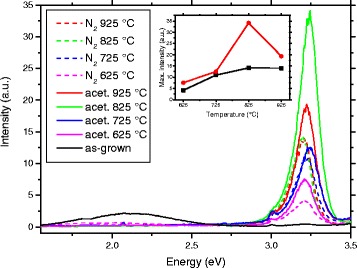
Room-temperature photoluminescence spectra of ZnO nanorod samples. The *inset* shows the temperature dependence of UV peak areas

In Fig. 8, we show the photoluminescence (PL) emission spectra from samples treated in various temperatures, measured with 337.1-nm laser excitation. The as-grown ZnO nanorods display no detectable photoluminescence in the UV range but produce a wide visible orange luminescence centered at 2.11 eV. In contrast, the visible peak in all thermally annealed samples is greatly diminished and the intensity of the UV peak strengthened. The UV peak in these samples, around 3.22 eV, is some 40 meV lower than what has been reported for free excitons, and may consist of excitonic signals in conjunction with their phonon replicas [38].

Various explanations for the visible range emission in ZnO have been proposed in the literature, though most interest has been in the green luminescence of ZnO. Considering the abundance of oxygen observed in our samples, the assignment to a zinc vacancy related complex (2.19 eV in [39]) seems most plausible. While Zn(OH)_2_ on the surface of the nanoparticles could also explain the quenching of the UV emission, we could not detect major changes in the O1s peak (Supporting Information), and thus do not believe that the dissociation of possible Zn(OH)_2_ could play a major role [40].

The results mean that the treatment has significantly reduced the amount of defects in the surface structure, as these are commonly associated with visible range photoluminescence emission [41]. The result is not surprising, as all ZnO point defects are expected to be mobile at the temperatures used in the treatments [42].

The inset of Fig. 8 shows the UV peak areas of the samples with respect to the treatment temperature. From here it can readily be seen that the acetylene-treated samples consistently display a stronger UV photoluminescence than those samples that were annealed in N_2_ at the same temperature. A maximum of difference can be seen at 825 °C treated samples, in which the difference in photoluminescence intensity is almost 2.5-fold. Compared to the untreated samples, the difference is over 60-fold. The same temperature also gives the strongest UV luminescence intensity for both types of samples.

**Fig. 9:**
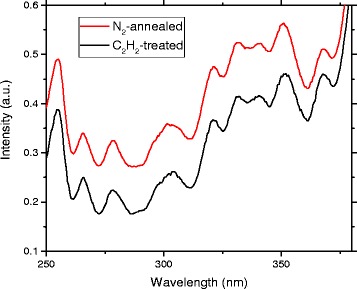
Photoemission excitation spectra for N_2_-annealed and acetylene-treated samples

Figure 9 shows the excitation spectra of samples treated in 825 °C for 410 nm emission. The spectra have been normalized to the intensity of the smallest wavelength maximum and shifted for clarity. No definite differences between the N_2_-annealed and acetylene-treated samples were observed, meaning that the observed changes in UV emission intensity are not due to differences in the above-bandgap absorption properties of the material.

### Raman Spectroscopy

**Fig. 10:**
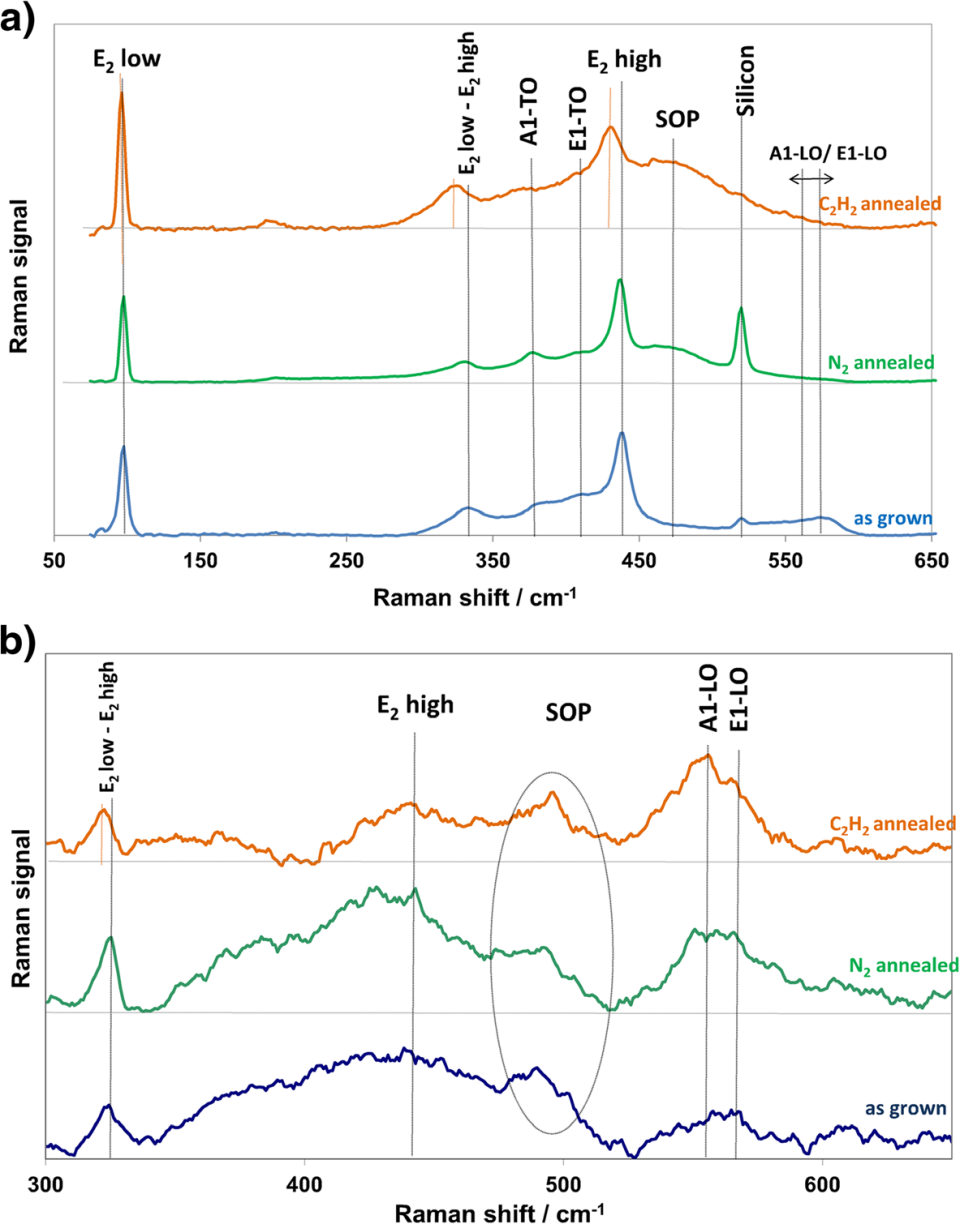
Raman spectra taken with excitation wavelengths of 532 nm (**a**) and 266 nm (**b**)

Figure 10 shows the Raman spectra from three ZnO samples. All samples show usual ZnO vibrational modes, as indexed in Fig. 10. The absence of the localized vibrational mode (LVM) band in 532 nm (Fig. 10a) excitation spectra at around 270 cm^−1^ implies that there is no O-substituted nitrogen in the samples, as this LVM band has been attributed to such defects [43]. The FWHM value of the E2 low peak is 8.3 cm^−1^ in the as-grown sample but is reduced to 7.2 and 7.4 cm^−1^ in the nitrogen-annealed and acetylene-treated samples, respectively. This shows that heat treatment reduces disorder in the zinc sublattice, as may be expected. In addition, the surface optical phonon mode (SOP), located at about 490 cm^−1^, is more intense in the acetylene-treated sample, which implies that the density of surface defects is bigger in the acetylene-treated sample, even though PL results implicate that the total number of defects, bulk included, must be reduced [44, 45]. This observation corresponds with the TEM images, which show a rougher surface in the samples treated with acetylene.

In the surface-sensitive 266-nm spectra (Fig. 10b), a wide and weak E2 peak (characteristic of wurtzite ZnO [15]) can be observed at 437.5 cm^−1^, as opposed to the sharper features in the bulk-related 532-nm spectra. This suggests the existence of surface disorder in the samples. The E2 high peak is associated with the oxygen sublattice, so the results are indicative of an oxygen-related disorder in all the rods studied here. The signal is weaker in the acetylene-treated rods compared to N_2_-annealed ones. This is likely to result from the etching and additional disorder in the surface as seen in the SEM images, most likely as the result of carbon incorporation into the lattice. The presence of A1 and E1 longitudinal optical (LO) optical modes, especially strong in the 266-nm spectra, further hints at disorder in the surface region [46].

The difference between the surface and bulk Raman measurements shows that the surfaces of the rods have more defects than the cores, which is likely related to the particle-like surface structure. The FWHM of the E2 low peak is 14.9 cm^−1^ in the as-grown sample but is reduced to 9.67 cm^−1^ by N_2_ annealing. In the acetylene-treated sample, the value is between the two, 11.3 cm^−1^, showing that while heat treatment eliminates disorder in the surface, additional disorder results as a result of etching and carbon atoms on the surface. As this is not seen in the 532-nm spectra, it can be surmised that the changes are concentrated on the surface of the rods.

### Discussion

Unlike in the previous work [18], the acetylene treatment was found to have only a limited effect on the morphology of the ZnO rods. However, some etching was still evident in the SEM images, and the effects of the etching are likely to be more dominant should smaller nanorods be used. To understand the differences between different types of ZnO samples, we should consider a possible mechanism for the etching.

It is known that ZnO can react with carbon in order to produce zinc vapor. The reaction proceeds as

1 $$ \mathrm{Z}\mathrm{n}\mathrm{O} + \mathrm{C}\to \mathrm{Z}\mathrm{n} + \mathrm{C}\mathrm{O} $$

with decomposing C_2_H_2_ providing the carbon. A two-step reaction consisting of the subreactions

2 $$ \mathrm{Z}\mathrm{n}\mathrm{O} + \mathrm{C}\mathrm{O}\to \mathrm{Z}\mathrm{n} + \mathrm{C}{\mathrm{O}}_2 $$

and

3 $$ \mathrm{C}{\mathrm{O}}_2 + \mathrm{C}\to 2\mathrm{C}\mathrm{O} $$

has also been considered, with direct oxidation of carbon monoxide to dioxide also possibly competing with reaction (2) [47]. While the boiling point of zinc, 907 °C, lies between the temperatures used in this study, any liquid zinc is also likely to be quickly evaporated and carried away by the gas flow due to the high temperatures and minuscule amounts involved [48].

The latter mechanism requires oxygen for the formation of CO. In our experiments, the tube was flushed for at least 20 min before treatment with N_2_, followed by 15-min flushing with 1:1 acetylene/N_2_. It seems unlikely that an appreciable amount of residual oxygen could be found in the tube after flushing. The oxygen for CO formation would then most likely come from the surfaces of the nanorods. In the temperature used, any excess oxygen can easily migrate from the structure and come in contact with adsorbed and/or decomposed C_2_H_2_ on the surface to produce CO, and the etching is controlled by the availability of oxygen. It must be noted, however, that the detected Zn/O ratio is the smallest in acetylene-treated samples. Additional CO reactions with the remaining ZnO, as expected in some gas sensing studies of ZnO, could be involved in the process, allowing one oxygen atom to vaporize more than one zinc atom [49].

While one would normally associate the enhanced UV luminescence found in the acetylene-treated rods to the reduction of visible range emissions, as the two processes compete with each other, it is insufficient to explain the effect observed in our study, as the visible range emission is already strongly reduced by the thermal treatment, with no further reduction by the acetylene treatment observed. Thus, is seems likely that the increased luminescence intensity is a result of a lowered possibility of non-radiative, rather than radiative, visible recombination of excitons in the acetylene-treated rods. The nature of non-radiative traps in ZnO is not completely clear, but based on the Raman results, it seems likely that changes in phonon dynamics in the oxygen sublattice may be the cause for the enhancement. The exact mechanism behind the phenomenon warrants further study.

## Conclusions

We have used the ACG process to produce ZnO nanorods and studied the effect of a thermal acetylene treatment on their structural and optical properties. The process was found to erode the surfaces of the nanorods, likely through a reaction of carbon from the acetylene and ZnO. In contrast to previous studies, the erosion was found to be limited, showing that the growth method has a great impact on the results of the treatment.

Acetylene treatment was found to remarkably increase the UV photoluminescence efficiency of the nanorods by up to a factor of 2.5 compared to N_2_-annealed ones, and with a 60-fold increase compared to as-grown rods. A treatment temperature of 825 °C was optimal for maximizing the effect. The main cause is proposed to be the suppression of non-radiative relaxation pathways on the surface of the nanorods due to the disruption of the oxygen sublattice by carbon species, rather than reduction of defect states. The improvement of luminescence may prove useful in future ZnO-related applications, such as nanoscintillators.

## Additional file

Additional file 1: Supporting information: XPS O1s peaks from ZnO nanorod samples. (PDF 404 kb)

## Abbreviations

ACG: Aqueous chemical growth
DI water: Deionized water
FWHM: Full width at half maximum
LO phonon: Longitudinal optical phonon
LVM: Localized vibrational mode
SEM: Scanning electron microscope
TEM: Transmission electron microscope
PL: Photoluminescence
SAED: Selected area electron diffraction
SOP: Surface optical phonon
UV: Ultraviolet
XPS: X-ray photoelectron spectroscopy
XRD: X-ray diffraction
ZnO: Zinc oxide
