# Supplementary material for: Enhanced Photoluminescence in Acetylene-Treated ZnO Nanorods
Source: Nanoscale Res Lett. 2016 Sep 20;11:413. doi: 10.1186/s11671-016-1627-y (PMC5028353; doi:10.1186/s11671-016-1627-y)
Supplement: Additional file 1: — Supporting information: XPS O1s peaks from ZnO nanorod samples. (PDF 404 kb) [file 11671_2016_1627_MOESM1_ESM.pdf]

Supporting information for ”Enhanced photoluminescence in acetylene-treated ZnO nanorods” by Luke Jäppinen et al.

Figures S1–S3 consist of XPS O1s peaks for untreated, nitrogen-annealed and acetylene-treated ZnO nanorods. B.E. scales are not calibrated: as-grown and N_2-annealed B.E.s 0.7 eV to large, acetylene-treated 1.5 eV too large (relative to the adventitious carbon peak at 284.8 eV)

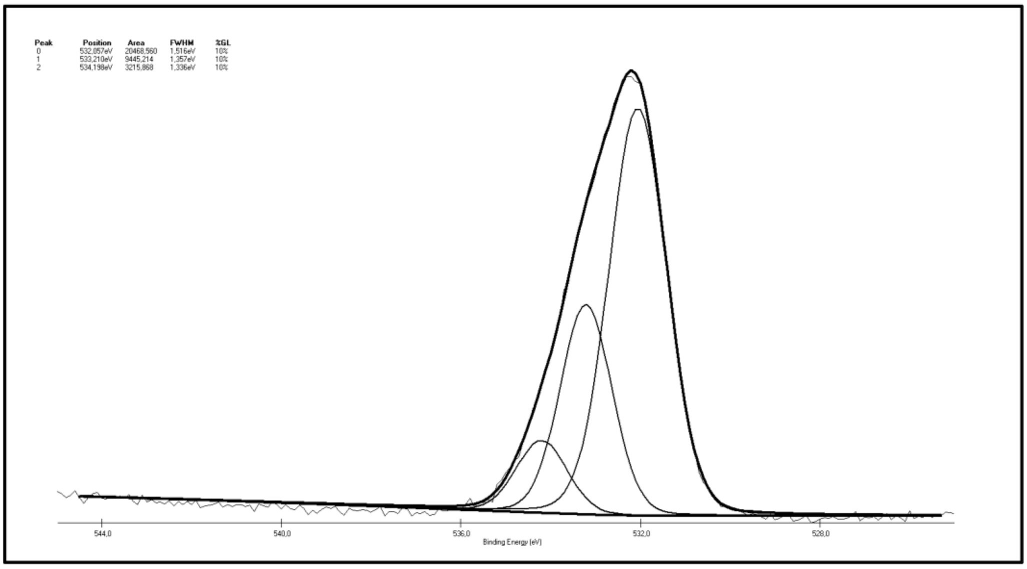

Figure S1: Untreated O1s

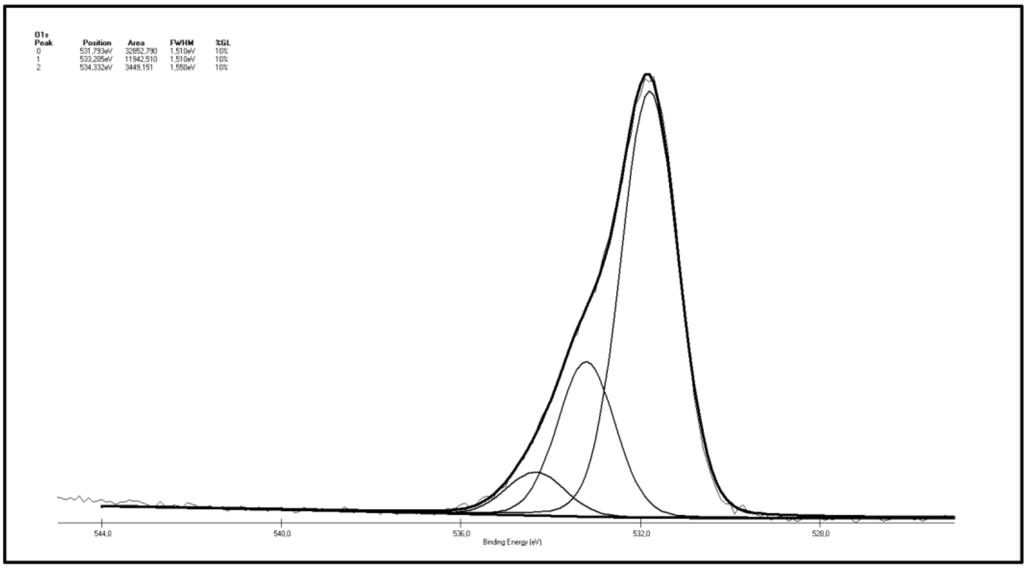

Figure S2: N_2-annealed O1s

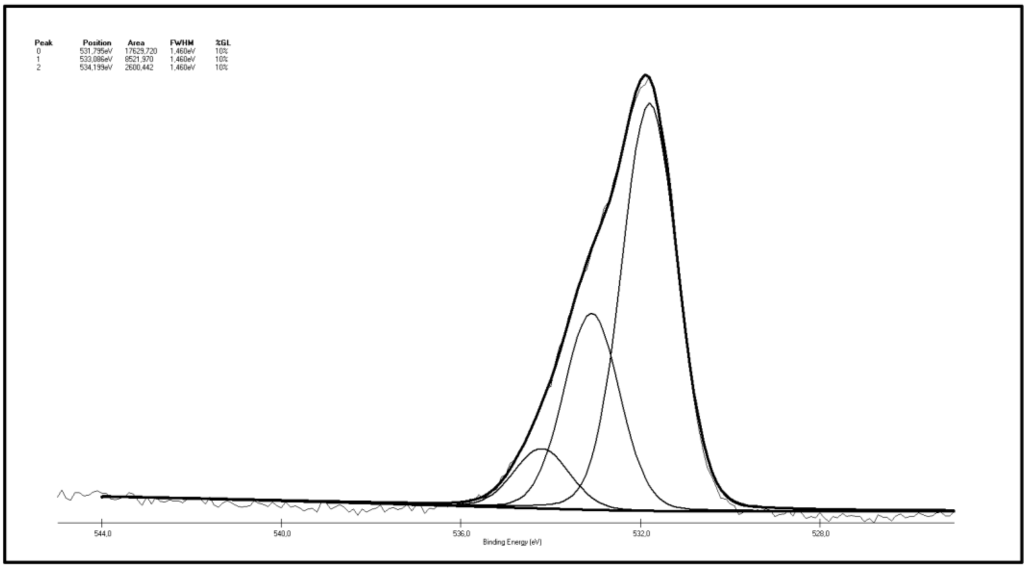

Figure S3: Acetylene-treated O1s
